# Transcription Factor-Based Biosensor for Dynamic Control in Yeast for Natural Product Synthesis

**DOI:** 10.3389/fbioe.2021.635265

**Published:** 2021-02-05

**Authors:** Yiming Zhang, Shuobo Shi

**Affiliations:** Beijing Advanced Innovation Center for Soft Matter Science and Engineering, College of Life Science and Technology, Beijing University of Chemical Technology, Beijing, China

**Keywords:** yeast, biosensor, natural products, fatty acids, shikimate pathway, MEP/MVA pathway, transcription factor

## Abstract

The synthesis of natural products in yeast has gained remarkable achievements with intensive metabolic engineering efforts. In particular, transcription factor (TF)-based biosensors for dynamic control of gene circuits could facilitate strain evaluation, high-throughput screening (HTS), and adaptive laboratory evolution (ALE) for natural product synthesis. In this review, we summarized recent developments of several TF-based biosensors for core intermediates in natural product synthesis through three important pathways, i.e., fatty acid synthesis pathway, shikimate pathway, and methylerythritol-4-phosphate (MEP)/mevalonate (MVA) pathway. Moreover, we have shown how these biosensors are implemented in synthetic circuits for dynamic control of natural product synthesis and also discussed the design/evaluation principles for improved biosensor performance.

## Introduction

Microbial synthesis of natural products not only ensures sustainable production but also enables synthesis of novel derivatives of interest. Yeast has been exploited as microbial cell factories to produce natural products that belong to several families, including fatty acids, isoprenoids, flavonoids, and alkaloids (Meadows et al., 2016; Rodriguez et al., 2017; Yu et al., 2018; Srinivasan and Smolke, 2020). With good knowledge of cell metabolism and well-developed synthetic biology technology and tools, much progress has been made through intensive metabolic engineering efforts to maximize flux toward natural products and improve titers, rates, and yields (Cravens et al., 2019; Liu et al., 2019, 2020). However, efficient biosynthesis is still challenging, and metabolic imbalance might be a key issue accounting for low yields and titers. Recently, dynamic control strategies have been developed to address the imbalance issue, as well-reviewed in Shen et al. (2019); Hartline et al. (2020), and Marsafari et al. (2020). Among these strategies, transcription factor (TF)-based biosensors could regulate the expression of gene circuits in response of specific intracellular metabolites and have been applied in natural product synthesis (Hossain et al., 2020). Besides, biosensors could accelerate metabolite quantification and have been used for strain evaluation, high-throughput screening (HTS), and adaptive laboratory evolution (ALE) (Williams et al., 2016; Qiu et al., 2019; Hossain et al., 2020).

In a TF-based biosensor, TF undergoes an allosteric conformational change induced by metabolite binding, which affects its binding at its operator and thereof regulates gene transcription (Zhang and Keasling, 2011; Zhang et al., 2015). Among a wide range of natural TFs that have been utilized in yeast, prokaryotic TFs have attracted much attention due to its relatively simple transcriptional regulation mechanism compared to eukaryotic TFs. A prokaryotic TF can be translated into a functional yeast biosensor with its operator inserted into a well-characterized promoter or a synthetic promoter (Qiu et al., 2019; Wan et al., 2019). Besides, endogenous TFs could potentially be utilized in yeast biosensor, as demonstrated in several studies (Zhang et al., 2016; Leavitt et al., 2017). For better performance in dynamic control of gene circuits, a TF-based sensor is required to be orthogonal and tunable in its dynamic range, operational range, specificity, and sensitivity, considered as common design and evaluation principles (Hossain et al., 2020).

**FIGURE 1:**
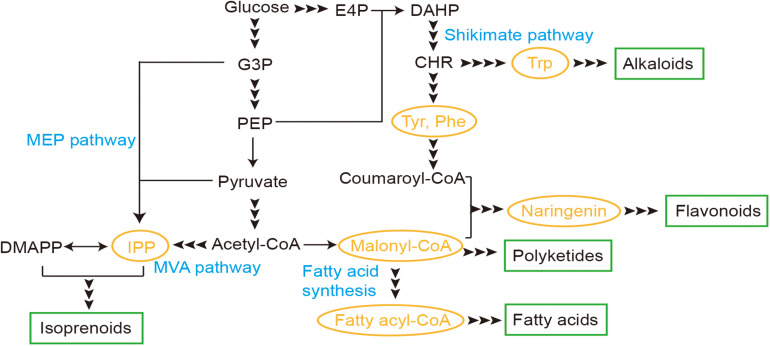
Transcription factor (TF)-based biosensors involved in synthesis pathways of natural products in yeast. E4P, erythrose-4-phosphate; G3P, glyceraldehyde-3-phosphate; PEP, phosphoenolpyruvate; IPP, isopentenyl pyrophosphate; DMAPP, dimethylallyl pyrophosphate; DAHP, 3-deoxy-D-arabino-2-heptulosonic acid 7-phosphate; CHR, chorismic acid; MEP, methylerythritol-4-phosphate; MVA, mevalonate; Trp, tryptophan; Tyr, tyrosine; Phe, phenylalanine.

Here we reviewed several TF-based biosensors developed in yeast and recent applications for production of natural products, including biosensors for malonyl-CoA, fatty acyl-CoA, isopentenyl pyrophosphate (IPP), naringenin, and aromatic amino acids (AAAs), which are core metabolites for synthesis of several families (Figure 1). Through these studies on the biosensors, design and evaluation principles were also reviewed and discussed, as well as engineering strategies for improved performance.

## Sensors Developed for Dynamic Control of the Fatty Acid Synthesis Pathway

The fatty acid synthesis pathway from malonyl-CoA via a cyclic elongation manner could be engineered and utilized for production of natural products like cocoa butter equivalents and jojoba-like wax esters (Bergenholm et al., 2018; Wenning et al., 2019; Figure 1). Biosensors for two essential intermediates malonyl-CoA and fatty acyl-CoA have been developed and discussed as below.

### Malonyl-CoA Biosensor

**FIGURE 2:**
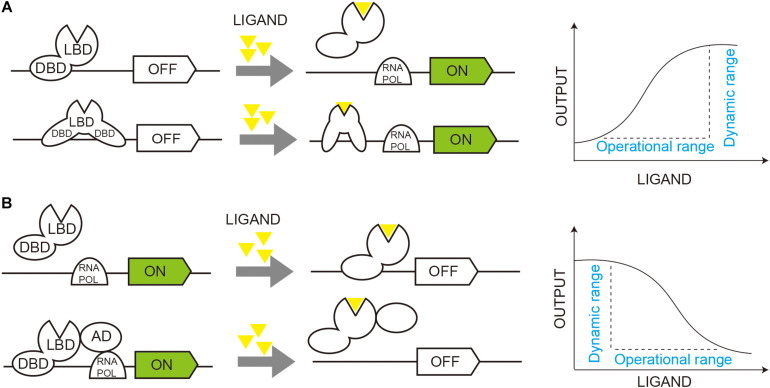
Dynamic regulation of gene circuits through TF-based biosensors. **(A)** Activated regulation of gene circuits. Transcription factor (TF) functions as a repressor to inhibit gene transcription (OFF), and ligand binding induces a conformational shift in TF and activates gene transcription (ON). **(B)** Repressive regulation of gene circuits. TF functions as an activator of gene transcription (ON), and ligand binding induces a conformational shift in TF and inhibit gene transcription (OFF). DBD, DNA binding domain; LBD, ligand binding domain; RNA Pol, RNA polymerase.

Malonyl-CoA serves as a basic unit for fatty acids, flavonoids, and non-ribosomal polyketides. The fatty acid and phospholipid regulator FapR and its operator *fapO* from *Bacillus subtilis* have been extensively studied and exploited for malonyl-CoA sensors in *E. coli*, *S. cerevisiae*, and mammalian cells (Ellis and Wolfgang, 2012; Xu et al., 2014a; Li et al., 2015; Johnson et al., 2017). FapR binds to *fapO* and functions as a repressor to inhibit gene transcription, and the repression can be relieved by malonyl-CoA, which induces a conformational shift in FapR and releases it from *fapO* (Schujman et al., 2006; Xu et al., 2014b; Figure 2A).

In yeast, FapR was usually expressed under a strong constitutive promoter to regulate reporter gene expression under a *fapO*-hybrid promoter, in which *fapO* was inserted in a well-characterized promoter. Using fluorescence proteins as the reporters, different designs were evaluated for sensitivity, dynamic range, and operational range (Li et al., 2015; David et al., 2016; Chen et al., 2018), including varying FapR/*fapO* ratio or *fapO* position in hybrid promoters and introducing nucleus localization sequences (NLS).

With a dual plasmid sensor system, one harboring FapR expressed under a *TEF1* promoter and the other harboring tdTomato regulated under a hybrid *fapO-GPM1* promoter, Li et al. (2015) found that a strong SV40 NLS significantly enhanced FapR nuclear import and subsequent repression. The biosensor with multicopy FapR and single *fapO* was identified as a better design with a broad dynamic range, up to 4-fold fluorescence increase in response to 8 mg/L cerulenin, which was supplemented as a fatty acid synthase inhibitor to increase the cellular malonyl-CoA level (Li et al., 2015). In another study, David et al. (2016) established a malonyl-CoA sensor with FapR expressed under a *TEF1* promoter and a green fluorescent protein (GFP) under a hybrid *fapO-TEF1* promoter. The optimized sensor with three *fapO* sites could respond to 13.5 μM cerulenin with up to 1.9-fold increase in the GFP fluorescence. The biosensor was then utilized for dynamic control for 3-hydroxypropionic acid (3-HP) production with fatty acid synthase expressed under a glucose sensitive *HXT1* promoter to control the malonyl-CoA availability. The hierarchical dynamic control improved 3-HP titer from 0.4 to 1.0 g/L, which could be applied for production of other malonyl-CoA-derived natural products. Ferreira et al. (2019) also combined the sensor with dCas9-based regulation for fine-tuned metabolism to increase acetyl-CoA and malonyl-CoA availability, which facilitated HTS and significantly improved 3-HP production. In a recent study, a malonyl-CoA sensor was constructed with FapR expressed under a *TEF1* promoter and yeGFP (yeast-enhanced GFP) under a hybrid *TEF1-fapO-GAL1* promoter and showed a better sensitivity, up to 8-fold yeGFP increase in response to 10 μM cerulenin (Chen et al., 2018). The sensor was then used to screen phosphorylation site mutations of acetyl-CoA carboxylase Acc1 and identified that Acc1^*S*686A*S*659A*S*1157A^ could benefit malonyl-CoA and 3-HP production.

Interestingly, when FapR was fused with transcriptional activation domain (AD) of an activator, it activated gene transcription under a *fapO*-hybrid promoter (Qiu et al., 2020; Figure 2B) instead of repressing. Qiu et al. (2020) compared several activators, including Gal4 AD, herpes simplex virus VP16, yeast transcriptional mediator Med2, and hybrid activators Med2-Gal4 and VP64-p65-Rta (VPR), and identified Med2 as the best candidate, which activated transcription by more than 40-fold, compared with the control without FapR-AD. With an optimized hybrid promoter, the *LEU2* promoter with the 1 *fapO* site inserted, the malonyl-CoA sensor increased GFP intensity by 53-fold compared to the control without FapR-AD. The final sensor showed a dose-dependent repressive response to cellular malonyl-CoA in an operational range of 0–20 μM cerulenin.

### Fatty Acyl-CoA Biosensor

Fatty acyl-CoAs serve as direct precursors for fatty acid-derived bioproducts (Marchetti et al., 2018). Transcription factor FadR and its operator *fadO* from *E. coli* have been evaluated for fatty acyl-CoA sensors in yeast (Teo et al., 2013; Teo and Chang, 2014; Dabirian et al., 2019). In *E. coli*, FadR regulates fatty acid metabolism with dual functions, which represses essential genes of fatty acid degradation and activates those of fatty acid synthesis by binding different regions of corresponding promoters (van Aalten et al., 2001; Iram and Cronan, 2005).

Teo et al. (2013) first established a FadR-*fadO* sensor in yeast with FadR expressed under a *TEF1*/*CYC1* promoter and yEGFP under a hybrid *fadO-GAL1* promoter, in which FadR functioned as a repressor. Biosensor optimization with varied FadR/*fadO* ratios identified that a combination of a *TEF1* promoter and three operators resulted in a sensor with a broad dynamic range, which could respond to 1 mM extracellular myristic acid with 1.4-fold induced transcription (Teo et al., 2013). Dabirian et al. (2019) also tuned FadR expression and modified hybrid *fadO-TEF1* promoters to optimize the biosensors. The optimized biosensor was then utilized in HTS of a gene overexpression library to increase the fatty acyl-CoA pool. The enriched genes were then evaluated for fatty acid and fatty alcohol production and identified three genes that could increase fatty alcohol levels by 1.8-fold (Dabirian et al., 2019). In another study, Teo and Chang (2014) combined the FadR-*fadO* biosensor with inducible promoters to construct AND-gate dynamic controllers, in which enhancer sequences of inducible promoters (*CUP1*, *PHO5*) were fused to a synthetic *GAL1* core promoter containing three *fadO* sites. In the AND-gate controllers, both fatty acids and copper presence/phosphate starvation were required to switch the AND-gate ON (Teo and Chang, 2014). The successful combination of logic gates with a biosensor would represent more flexible dynamic control on gene circuits for bioproduct synthesis in yeast.

By fusing AD to FadR, the FadR repressor could be translated to a transcriptional activator in the presence of the effector molecule acyl-CoA (Qiu et al., 2020). When FadR was fused with VPR and Med2, the biosensor resulted in transcriptional activation by 23.9- and 28.4-fold, respectively. The engineered biosensor with FadR-Med2 and one *fadO* downregulated gene expression to 42.8% as 2.0 mM oleic acid was supplemented.

## Sensors Developed for Dynamic Control of the MVA/MEP Pathway

Isoprenoids were synthesized via carbon chain elongation by repeated addition of IPP or dimethylallyl pyrophosphate, which could be produced either from acetyl-CoA via the MVA pathway or from pyruvate and glyceraldehyde-3-phosphate through the MEP pathway (Figure 1). IPP biosensors have been developed and engineered for its potential application in isoprenoid synthesis.

Isopentenyl pyrophosphate (IPP) is an essential intermediate for isoprenoid synthesis, and its supply may be limited to its high toxicity. Therefore, dynamic control through the IPP node will benefit synthesis pathways for isoprenoids. However, no natural TF has been identified responsive to IPP. In yeast, a synthetic TF was repurposed for sensing IPP by fusing IPP isomerase Idi to the *GAL4* AD and DNA binding domain (DBD), respectively. With Idi-DBD bound to the *GAL10* promoter, Idi dimerization triggered by IPP could bring Idi-AD close enough to activate transcription of a fluorescent protein yEcitrine (Chou and Keasling, 2013). The IPP biosensor was introduced into a reported isoprenoid-producing strain MO219 (Ro et al., 2006) and showed 1.5-fold increase in fluorescence when its MVA pathway overexpression was induced to increase IPP supply. The authors additionally replaced Idi with another two enzymes utilizing IPP as a substrate (Idi1 and Erg20), and the two sensors showed higher fluorescence levels upon galactose induction. The strategy to construct IPP sensors with synthetic TFs was demonstrated applicable in *E. coli* as well and was used in a feedback-regulated evolution to improve lycopene production by nearly 6.8-fold. Therefore, synthetic TFs alleviated the need to rely on preexisting biological components.

## Sensors Developed for Dynamic Control of the Shikimate Pathway

The shikimate pathway for biosynthesis of AAAs has been studied and engineered for *de novo* biosynthesis of various aromatic products, like flavonoids and alkaloids (Figure 1). Here, we focused on biosensors developed for core intermediates, including naringenin and AAAs.

### Naringenin Biosensor

Naringenin can be synthesized from malonyl-CoA and coumaroyl-CoA derived from intermediates of the shikimate pathway and is a key intermediate for synthesis of other flavonoids used as nutritional supplementary and pharmaceuticals (Figure 1). Transcription factor FdeR from *Herbaspirillum seropedicae* and its operator *FdeO* were repurposed as naringenin biosensors in both *E. coli*, *S. cerevisiae* and *Yarrowia lipolytica* (Skjoedt et al., 2016; Lv et al., 2020).

In *S. cerevisiae*, a naringenin biosensor was constructed via expressing FdeR under a *TDH3* promoter and GFP under a hybrid *FdeO-CYC1* promoter (Skjoedt et al., 2016), in which FdeR functioned as a co-inducer of naringenin. The sensor, which could induce 1.7-fold GFP increase in response to 0.2 mM naringenin, was then transformed into engineered strains to monitor naringenin production. Strong linear correlations at 24 h (*r* = 0.87) and 48 h (*r* = 0.96) between GFP intensities and metabolite concentrations (Skjoedt et al., 2016) made the sensor applicable for HTS of naringenin production strains. In a recent study, by varying NLS locations and expression levels, a naringenin sensor with NLS-FdeR expressed under a *TDH3* promoter and mCherry under a hybrid *FdeO-GPM1* promoter in a high-copy number plasmid showed a relatively broad dynamic range and sensitivity, a 3-fold fluorescence increase in response to 0.2 mM naringenin. The sensor was then to screen a modular assembled naringenin biosynthetic library with 972 combinations and obtained a strain with a titer of 52.0 mg/L naringenin (Wang et al., 2019).

In *Y. lipolytica*, a naringenin biosensor with FdeR expressed under a *TEF* promoter and the *Nluc* luciferase under a hybrid *Fdeo-TEF* promoter was constructed, and the sensor showed an operational range of 0–50 mg/L naringenin (Lv et al., 2020). By expressing FdeR under a weak promoter and Leu2 under a hybrid *Fdeo-TEF* promoter, the sensor enabled naringenin inducible growth in a leucine auxotrophic strain. The modified sensor was found to selectively enrich the naringenin-producing population and maintain strain stability (Lv et al., 2020).

### Aromatic Amino Acid Biosensor

Aromatic amino acids (AAAs) including tryptophan (Trp), tyrosine (Tyr), and phenylalanine (Phe) are synthesized from the intermediates of the shikimate pathway (Figure 1). Endogenous regulation induced by AAAs in yeast has been studied and considered as attractive and potent inducible for metabolic engineering. Expression of *ARO9*, encoding AAA transferase II protein, was found activated via transcription factor Aro80 in the presence of AAAs (Lee and Hahn, 2013), which could be exploited for development of AAA biosensors.

Leavitt et al. (2016) enlarged the minimally sufficient *UAS*_*aro*_ element dissected from the *Aro9* promoter with Aro80 binding site and fused it with a core promoter to form a hybrid promoter, which could induce the expression of yellow fluorescent protein (YFP) in response to exogenous tryptophan. With an Aro80 variant expressed under a strong *GAL1* promoter and five *UAS*_*aro*_ sites inserted in a minimal core promoter, a AAA biosensor was constructed, which could respond to 1 g/L tryptophan and 1 g/L galactose with 6- and 12-fold induced expression, respectively. In the presence of tryptophan and galactose, the biosensor resulted in 14-fold induced GFP expression. This work demonstrated the potential of native transcription factors for biosensor construction.

A biosensor based on Aro80-*UAS*_*aro*_ was then used in ALE for production of AAAs and muconic acid (Leavitt et al., 2017). In the biosensor, *YFP* was replaced by the antibiotic gene *KanNeo* under a hybrid *UAS*_*aro*_-*LEU* promoter to couple cell growth with AAA production. Based on the anti-metabolite selection with 4-fluorophenylalanine in ALE, the strains were evolved with up to 2-fold higher total AAA production, which were engineered to redirect increased flux to muconic acid production by expressing a truncated Aro1 and an aromatic decarboxylase. The final strain could produce 0.5 g/L muconic acid in shake flasks and 2.1 g/L in a fed-batch bioreactor.

Prokaryotic TF was also exploited for tryptophan biosensors in yeast. TrpR from *E. coli* functions as an aporepressor, and it binds at its operator *TrpO* in the presence of tryptophan to repress transcription (Gunsalus and Yanofsky, 1980; Figure 2B). In yeast, a tryptophan biosensor was developed based on TrpR-*TrpO* with *GFP* expressed under a hybrid *TrpO-TEF1* promoter (Zhang et al., 2020). The biosensor could repress GFP expression by up to 2.4-fold in an operational range of 2–200 mg/L tryptophan. The repression biosensor was then converted to an activation sensor by fusing Gal4 AD to TrpR (Gal4_*A*__*D*_-TrpR), which was expressed under a weak *REV1* promoter. With six copies of *TrpO* inserted into a *GAL1* core promoter, the constructed biosensor could sense 2–200 mg/L tryptophan with a 5-fold dynamic range. The biosensor was used for HTS of a combinatorial library based on a platform strain with increased AAA accumulation and facilitated mechanistic and machine learning models with the recommendations, which improved tryptophan titer and productivity by up to 74 and 43%, respectively (Zhang et al., 2020).

## Conclusion and Perspectives

Transcription factor (TF)-based biosensors have become powerful tools in strain evaluation, HTS, and ALE for yeast synthesis of natural products. With more natural TFs identified and characterized both experimentally and bioinformatically, TF-based biosensors for more metabolites could be established to facilitate natural product synthesis. However, one big challenge for TF biosensors might be for their applicability in natural product synthesis where a larger dynamic range is needed. The biosensor design may need to be adjusted or evolved as the titers become higher or be matched to the production kinetics of the individual strain or library of biocatalysts.

Biosensor engineering on TFs and the hybrid operator-promoters have enabled improved sensing sensitivity, dynamic range, operational range, and ligand specificity, as well as inversion of function between activated and repressive regulation (Skjoedt et al., 2016; Snoek et al., 2019; Ambri et al., 2020; Qiu et al., 2020). Evolution-guided engineering equipped with TF mutagenesis and user-defined FACS-based toggled selection could be a versatile and high-throughput method to generate user-defined biosensors (Snoek et al., 2019). Also, the variants of a TF could be targeted for new effectors with the help of protein engineering as well as computational design (de los Santos et al., 2016). Optimal biosensor reporter promoter scanning revealed that TF operator positions could be critical for improved biosensor performances, enabling a redesigned TF biosensor with a dynamic output range up to 26-fold (Ambri et al., 2020). The functional inversion of TF-based biosensors allows for two distinct regulatory responses to the ligand metabolites and could be utilized to construct metabolite switch, as demonstrated in *E. coli*, which resulted in a balanced metabolism between cell growth and product formation (Xu et al., 2014a).

It is also interesting to notice the construction of logic gates in TF-based biosensors (Teo and Chang, 2014; Leavitt et al., 2016), which could be possibly exploited for higher-order designs in reprogramming dynamic regulation of gene circuits for natural product synthesis. Besides, G-protein-coupled receptor (GPCR)-based biosensors have been engineered for sensing medium-chain fatty acids in yeast (Mukherjee et al., 2015), representing alternative genetic biosensors to dynamically control the synthesis of natural products in yeast (Marsafari et al., 2020). Additionally, the emerging optogenetics-based sensors can be applied with any change in chemicals (Marsafari et al., 2020), giving great advantages to advance the construction of future yeast cell factory to dynamically control cellular metabolism or dissect cellular network function.

## Author Contributions

YZ and SS outlined this manuscript. YZ drafted the manuscript. SS revised the manuscript. Both authors contributed to the article and approved the submitted version.

## Conflict of Interest

The authors declare that the research was conducted in the absence of any commercial or financial relationships that could be construed as a potential conflict of interest.
